# Face masks to prevent transmission of respiratory infections: Systematic review and meta-analysis of randomized controlled trials on face mask use

**DOI:** 10.1371/journal.pone.0271517

**Published:** 2022-12-01

**Authors:** Hanna M. Ollila, Markku Partinen, Jukka Koskela, John Borghi, Riikka Savolainen, Anna Rotkirch, Liisa T. Laine

**Affiliations:** 1 Institute for Molecular Medicine Finland (FIMM), University of Helsinki, Helsinki, Finland; 2 Broad Institute of MIT and Harvard, Cambridge, MA, United States of America; 3 Center for Genomic Medicine, Massachusetts General Hospital and Harvard Medical School, Boston, MA, United States of America; 4 Department of Anesthesia, Critical Care and Pain Medicine, Massachusetts General Hospital, Boston, MA, United States of America; 5 Helsinki Sleep Clinic, Terveystalo Healthcare, Helsinki, Finland; 6 Department of Clinical Neurosciences, Clinicum, University of Helsinki, Helsinki, Finland; 7 Helsinki University and Helsinki University Hospital, Clinic of Gastroenterology Helsinki, Helsinki, Finland; 8 Lane Medical Library, Stanford University School of Medicine, Stanford, California, United States of America; 9 Swansea University, Swansea, United Kingdom; 10 Population Research Institute, Väestöliitto, The Family Federation of Finland, Helsinki, Finland; 11 University of Missouri, Columbia, MO, United States of America; University of Phayao, THAILAND

## Abstract

**Objectives:**

To examine the use of face mask intervention in mitigating the risk of spreading respiratory infections and whether the effect of face mask intervention differs in different exposure settings and age groups.

**Design:**

Systematic review and meta-analysis. We evaluated the risk of bias using the Cochrane Risk of Bias 2 tool (ROB2).

**Data sources:**

We searched PubMed, Embase, Cochrane Central Register of Controlled Trials, and Web of Science were searched for randomized controlled trials investigating the effect of face masks on respiratory infections published between 1981 and February 9, 2022. We followed the PRISMA 2020 guidelines.

**Eligibility criteria for selecting studies:**

We included randomized controlled trials investigating the use of face mask intervention in mitigating the risk of spreading respiratory infections across different exposure settings.

**Results:**

We identified 2,400 articles for screening. 18 articles passed the inclusion criteria for both evidence synthesis and meta-analysis. There were N = 189,145 individuals in the face mask intervention arm and N = 173,536 in the control arm, and the follow-up times ranged from 4 days to 19 months. Our results showed between-study heterogeneity (p < 0.0001). While there was no statistically significant association over all studies when the covariate unadjusted intervention effect estimates were used (RR = 0.977 [0.858–1.113], p = 0.728), our subgroup analyses revealed that a face mask intervention reduced respiratory infections in the adult subgroup (RR = 0.8795 [0.7861–0.9839], p = 0.0249) and in a community setting (RR = 0.890 [0.812–0.975], p = 0.0125). Furthermore, our leave-one-out analysis found that one study biased the results towards a null effect. Consequently, when using covariate adjusted odds ratio estimates to have a more precise effect estimates of the intervention effect to account for differences at the baseline, the results showed that a face mask intervention did reduce respiratory infections when the biasing study was excluded from the analysis (OR = 0.8892 [0.8061–0.9810], p = 0.0192).

**Conclusion:**

Our findings support the use of face masks particularly in a community setting and for adults. We also observed substantial between-study heterogeneity and varying adherence to protocol. Notably, many studies were subject to contamination bias thus affecting the efficacy of the intervention, that is when also some controls used masks or when the intervention group did not comply with mask use leading to a downward biased effect of treatment receipt and efficacy.

**Trial registration:**

PROSPERO registration number CRD42020205523.

## 1. Introduction

Pandemics such as the current COVID-19 pandemic are a scourge causing severe losses to life, health, and the economy [1,2]. COVID-19 is caused by SARS-CoV-2 (Severe Acute Respiratory Syndrome Coronavirus 2). The main mode of SARS-CoV-2 spread is through airborne transmission [3–7]. While vaccines have been developed and are being distributed and more treatment drugs are becoming available, non-pharmaceutical interventions (NPIs) remain an important tool for reducing the number of COVID-19 infections [8]. Thus, a better understanding of the efficacy and effectiveness of transmission-reducing public health measures with NPIs would help in developing strategies to prevent the emergence of novel, potentially more transmissible and deadly variants.

Face masks have long been widely used in health care settings, but before the COVID-19 pandemic, the use of face masks by the general public was perceived in the Western world as a relatively new policy tool in preventing person-to-person transmission on a global scale. Prediction models, however, suggest that universal use of face masks in public may have a substantial preventive impact on respiratory disease spread, even without perfect adherence to protocol, compliance, or the use of medical masks [9–11]. In addition, a pooled meta-analysis of 172 articles regarding the spread of respiratory infections showed that mask-wearing was consistently effective in preventing the spread of infections by SARS-CoV-2 and the beta-coronaviruses that cause severe acute respiratory syndrome, as well as the Middle East Respiratory Syndrome [8].

On the other hand, some studies have suggested that the results from randomized controlled trials on the efficacy of face mask use among the general public are merely suggestive and that the effects of face masks are unclear [12,13]. One challenge in a randomized controlled trial is a so called contamination bias which for face masks would mean non-adherence to the protocol in either the face mask intervention arm or the control arm [14]. Because studies typically estimate the intention-to-treat effect, non-compliance can lead to underestimating the magnitude of the estimated treatment effect of face mask use for a given randomized controlled trial. This may explain some of the unclear results of the effects of face mask use in the literature.

Our systematic review and meta-analysis aimed to examine the evidence from the randomized controlled trials of face masks in the context of respiratory infections or diseases that spread through similar mechanisms as COVID-19. Earlier systematic reviews and meta-analyses have investigated the effect of face masks in preventing respiratory infection transmissions by focusing on the efficacy of cloth masks [15] or the efficacy of face masks in health care and non-health care settings [16]. These meta-analyses have combined various types of studies, including randomized controlled trials, case-control studies, and cohort studies. Our systematic review and meta-analysis complement these previous studies by focusing solely on randomized controlled trials conducted in different exposure settings (hospital, household, and community) and age groups to study if the face mask intervention would yield different result if it focused only for adults or populations with adults and children. In addition, we examined how the type of setting, age, and non-adherence to the protocol (non-compliance) in treatment and control arms were associated with the effects of the intervention.

## 2. Methods

This systematic review was reported according to the Preferred Reporting Items for Systematic Reviews and Meta-Analyses [17]. Our review question was: For the individuals at risk of contracting respiratory infections, does a face mask intervention reduce the risk for respiratory infections of those individuals in the intervention arm compared with those individuals that are not part of the face mask intervention? Our review protocol was registered on PROSPERO in November 2020 (registration number CRD42020205523), and it was updated on May 12, 2022 and September 22, 2022.

### 2.1. Search strategy

We performed the searches using the Cochrane Central Register of Controlled Trials (CENTRAL), EMBASE, PubMed, and the Web of Science (science and social science citation index). In the working paper version of this paper, we followed a similar search strategy as in [18] which was updated significantly to the search strategy described in Table 1 on February 9, 2022. The search terms for each database are shown in Table 1.

**Table 1:** Search strategy in each database. We list the concept, synonym, and syntax for each database. In addition, we provide Emtree and MeSH terms when applicable.

Database	Concept	Synonym	MeSH	Syntax
Medline / Pubmed	Infection	Infection[TW]	”Influenza, Human”[MH]	(”Influenza, Human”[MH] OR “SARS Virus”[MH] OR “SARS-CoV-2”[MH] OR “Coronavirus Infections”[MH] OR Infection[TW] OR Influenza[TW] Infections[TW] OR “Covid-19”[TW] OR
		Infections[TW]	“SARS Virus”[MH]	“SARS-CoV-2”[TW] OR “Coronavirus”[TW] OR “Swine Flu”[TW] OR “severe acute respiratory syndrome”[TW] OR “Middle East Respiratory Syndrome”[TW]SARS[TW] OR MERS[TW])
		“Covid-19”[TW]	“Coronavirus infections”[MH]	
		“SARS-CoV-2”[TW]	“SARS-CoV-2”[MH]	
		“Coronavirus”[TW]		
		“Swine Flu”[TW]		
		“severe acute respiratory syndrome”[TW]		
		“Middle East Respiratory Syndrome”[TW]		
		SARS[TW]		
		MERS[TW]		
	Masks	Mask*[TW]	“Masks”[MH]	(“Respiratory Protective Devices”[MH] OR “Masks”[MH] OR Mask[TW] OR Masks[TW])
			“Respiratory Protective Devices”[MH]	
	RCTs	Random[TW]	“Controlled Clinical Trial”[PT]	(“Controlled Clinical Trial”[PT] OR “Randomized Controlled Trial”[PT] OR “Randomized Controlled Trials as Topic”[MH] OR Random[TW] OR “Randomized[TW])
		Randomized[TW]	“Randomized Controlled Trial”[PT]	
		“Clinical trial”[TW]	“Randomized Controlled Trials as Topic”[MH]	
			“Controlled Clinical Trial”[PT]	
Embase	**Concept**	**Synonym**	**Emtree**	**Syntax**
	Infection	Infection:ti,ab,kw	“Influenza”/exp	(“Influenza”/exp OR
		Infections:ti,ab,kw	“severe acute respiratory syndrome”/exp	“severe acute respiratory syndrome”/exp OR
		“Covid-19”:ti,ab,kw	“coronavirus disease 2019”/exp	“coronavirus disease 2019”/exp OR Influenza:ti,ab,kw OR Infection:ti,ab,kw OR
		“SARS-CoV-2”:ti,ab,kw		Infections:ti,ab,kw OR
		“Coronavirus”:ti,ab,kw		“Covid-19”:ti,ab,kw OR
		“Swine Flu”:ti,ab,kw		“SARS-CoV-2”:ti,ab,kw OR
		“severe acute respiratory syndrome”:ti,ab,kw		“Coronavirus”:ti,ab,kw OR
		“Middle East Respiratory Syndrome”:ti,ab,kw		“Swine Flu”:ti,ab,kw OR
		SARS:ti,ab,kw		“severe acute respiratory syndrome”:ti,ab,kw OR
		MERS:ti,ab,kw		“Middle East Respiratory Syndrome”:ti,ab,kw OR
				SARS:ti,ab,kw OR
				MERS:ti,ab,kw)
	Masks	Mask*:ti,ab,kw	“Masks”/exp	(“Masks”/exp OR Mask:ti,ab,kw OR Masks:ti,ab,kw)
	RCTs	Random:ti,ab,kw	“controlled clinical trial”/exp	(“controlled clinical trial”/exp OR Random:ti,ab,kw OR
		Randomized:ti,ab,kw		Randomized:ti,ab,kw OR Clinical Trial:ti,ab,kw)
		Clinical Trial:ti,ab,kw		
Cochrane	**Concept**	**Synonym**		**Syntax**
	Infection	Infection:ti,ab,kw		Infection:ti,ab,kw OR Infections:ti,ab,kw “Covid-19”:ti,ab,kw OR “SARS-CoV-2”:ti,ab,kw OR “Coronavirus”:ti,ab,kw OR “Swine Flu”:ti,ab,kw OR “severe acute respiratory syndrome”:ti,ab,kw OR “Middle East Respiratory Syndrome”:ti,ab,kw OR SARS:ti,ab,kw OR MERS:ti,ab,kw
		Infections:ti,ab,kw		
		“Covid-19”:ti,ab,kw		
		“SARS-CoV-2”:ti,ab,kw		
		“Coronavirus”:ti,ab,kw		
		“Swine Flu”:ti,ab,kw		
		“severe acute respiratory syndrome”:ti,ab,kw		
		“Middle East Respiratory Syndrome”:ti,ab,kw		
		SARS:ti,ab,kw		
		MERS:ti,ab,kw		
	Masks	Mask*:ti,ab,kw		(Mask:ti,ab,kw OR Masks:ti,ab,kw)
	RCTs	Random:ti,ab,kw		(Random:ti,ab,kw OR Randomized:ti,ab,kw OR “Clinical Trial”:ti,ab,kw)
		Randomized:ti,ab,kw		
		Clinical Trial:ti,ab,kw		
Web of science	**Concept**	**Synonym**		**Syntax**
	Infection	Infection:ti,ab		TI = (Infection OR Infections OR “Covid-19” OR “SARS-CoV-2” OR “Coronavirus” OR “Swine Flu” OR “severe acute respiratory syndrome” OR “Middle East Respiratory Syndrome” OR SARS OR MERS)) OR ALL = (Infection OR Infections OR “Covid-19” OR “SARS-CoV-2” OR “Coronavirus” OR “Swine Flu” OR “severe acute respiratory syndrome” OR “Middle East Respiratory Syndrome” OR SARS OR MERS
		Infections:ti,ab		
		“Covid-19”:ti,ab		
		“SARS-CoV-2”:ti,ab		
		“Coronavirus”:ti,ab		
		“Swine Flu”:ti,ab		
		“severe acute respiratory syndrome”:ti,ab		
		“Middle East Respiratory Syndrome”:ti,ab		
		SARS:ti,ab		
		MERS:ti,ab		
	Masks	Face Mask*:ti,ab		(face mask:ti,ab OR face Masks:ti,ab)
	RCTs	Random:ti,ab: Randomized:ti,ab		(Random:ti,ab OR Randomized:ti,ab

We limited our searches to randomized controlled trials on humans published between 1981 and February 9, 2022. We did not limit the searches by language. We updated our search results that were uploaded on Covidence (Veritas Health Innovation, Melbourne, Australia), and we kept the unique citations. Lastly, two independent reviewers screened the unique citations for inclusion in the meta-analysis.

### 2.2. Intervention, inclusion criteria, exclusion criteria, and study selection

**Intervention.** We included randomized controlled trials on humans that compared various types of face masks with various filtering capacities. Different mask types include FFP1 masks with 80% filtration capacity, FFP2 masks with no less than 94% filtration, FFP3 masks with 99% filtration capacity, surgical masks, and cloth masks.

**Inclusion and exclusion.** We included interventions that were executed in various settings, such as in health care, in a community, or in a household (Table 2). We included studies that had an entire text available (including pre-prints).

**Table 2:** Inclusion and exclusion criteria.

Inclusion criteria	Exclusion criteria
1. Randomized controlled trial. 2. General population, or health care personnel in risk of contracting infectious diseases. 3. Health care, community, and household settings: non-restrictive for setting. 4. Intervention type: face mask by any filtration type: FFP1, FFP2, FFP3, cloth mask, or surgical mask. 5. Control arm has been assigned to have no face mask. 6. Mask use may occur with or without hand hygiene or other measures (e.g., use education). 7. Risk estimate for infection is reported. 8. Included: All ages and gender. 9. Publication format. Whole text available, preprints included. 10. Language of original publication; Primary search in English. No exclusion for other languages. 11. Sample size and follow-up length did not have exclusion criteria.	1. Empirical qualitative study. 2. Quasi-experimental study. 3. Control arm has been assigned to use a face mask. 4. Values for control arm were not provided. 5. No full text available. 6. Study is made on animals or to studies transmission from animals to humans. 7. Studies that examine effect on psychological factors (e.g., empathy). 8. Studies that examine non-respiratory illnesses (eg. wound infection).

We excluded studies that did not examine respiratory infections. We also excluded studies that estimated compliance, or filtration of masks, in addition to excluding studies that compared different types of face masks to each other (in which the comparison arm was assigned the use of a face mask). We did not exclude any studies based on age and gender or have exclusion criteria based on sample sizes or follow-up periods.

The outcomes of interest were the number of respiratory infections in the treatment group and the control group infection with and without a face mask intervention and in different settings. Furthermore, we evaluated the adherence to intervention. We included two outcome summary statistics measuring the risk of an infection in our analysis. The first one is the covariate unadjusted risk ratio estimate for an infection, which we computed by using the documented numbers of infected and not infected in the intervention arm provided in the studies.

It is possible that omitted variables, such as age, gender, demographics, and vaccination status such as correlation of outcomes within households or other clusters affect the outcomes. Thus, our second outcome summary statistic was the covariate adjusted odds ratio estimate if it was estimated in the study. For the studies that did not have estimated covariate adjusted odds ratios, we calculated the odds ratio using the documented numbers of infected and not infected in each intervention arm provided in the study. We used covariate adjusted odds ratio estimates instead of covariate adjusted risk ratio estimates because almost none of the studies provided the latter. In Section 4.3., we discuss in detail the limitations of the different summary statistics (risk ratio, odds ratio) used in the meta-analyses.

**Study selection.** JB designed the search. Three authors (JB, HMO, and LTL) executed the search. Two authors (HMO and LTL) independently reviewed all the titles and abstracts to define the papers that could potentially be included in the systematic review. After this, the same two authors independently screened the articles using Covidence and determined whether they met the inclusion and exclusion criteria. Any disagreements between the two authors were resolved by discussion between them. The search strategy and included articles were validated by another author (MP). We report the findings in compliance with the PRISMA 2020 statement.

### 2.3. Data extraction

Two authors (HMO and LTL) independently extracted the data from the original articles. Extracted values were (1) study setting (year, location, population, age of individuals, if intervention population consisted only of adults or if the intervention population included adults and children); (2) intervention details (randomization level, follow-up length, type of intervention, other interventions, case or index case definition); (3) outcome measure of Influenza Like Illness (if available), outcome measures (number of infected and not infected per intervention arm, covariate adjusted odds ratio effect estimates with respective standard errors); (4) adherence to protocol (e.g., if the study reports non-compliance in treatment, if the study reports that individuals in the control group used a face mask); and (5) main findings for the effects of face mask intervention from the study in addition the conclusion of the paper and supporting evidence for the finding. Three other reviewers (MP, JK, and RS) checked the extracted data for errors.

We contacted the corresponding author of Aiello 2010 and 2012 [19,20] and Larson et al., [21] to confirm our interpretation of their data and also asked if they were able to provide us with the covariate unadjusted risk ratio for the source control estimates. Unfortunately, the data was no longer available. Furthermore, we contacted the authors of Abaluck et al., [22], and they provided us with the code and the instructions on how to estimate the covariate adjusted odds ratio estimates.

### 2.4. Risk-of-bias assessment

Two review authors (HMO and LTL) independently assessed the risk of bias using the Cochrane Risk of Bias 2 tool (ROB2) [23]. Any discrepancies were resolved by consensus. The risks were categorized as low risk, some concern or high risk of bias.

### 2.5. Data analysis

The results for all the outcomes were expressed as risk ratio estimates (RR), odds ratio estimates (OR), and 95% confidence intervals for the effect estimates. We combined the estimates using a random-effects meta-analysis, based on the assumption that it was likely that methodological and clinical heterogeneity affected the results. We estimated the between-study variance by using the DerSimonian and Laird method of moments -estimator. We calculated the 95% confidence intervals using the Wald method. Furthermore, to account for differences at the baseline that were present despite the randomization, a subset of papers had provided covariate and intracluster correlated adjusted odds ratio estimates of the intervention effect. Therefore, the adjusted analyses are shown in odds ratio scale.

We assessed the homogeneity of the study-specific effect sizes using the ξ^2^ test and quantified statistical inconsistency by using the I^2^ statistic [24]. We used a subgroup analysis to explore heterogeneity in the estimates of the face mask effects according to the following: source control, adherence to protocol, study populations, and study settings. We ran meta-regressions to evaluate how the type of setting, age, non-compliance to treatment (not using a face mask despite being in the treatment arm) or non-compliance to control (using a face mask despite being in the control arm) was associated with the effects of the intervention. Finally, we computed meta-analyses with leave-one-out analysis to account for a potential bias created by any single study.

We evaluated the small study effects by generating a contour-enhanced funnel plot to examine the bias in the results of the meta-analysis as intervention effects from smaller studies tend to differ from those estimated in larger ones, which can result from reporting biases, methodological or clinical heterogeneity, or other factors.

We conducted all our analyses using the *meta*, *metafor*, *nnt*, and *dmetar* packages in R version 4.02 and using *meta* in Stata version 17.

### 2.6. Patient and public involvement

Our study did not involve patients or public participants because we used secondary data. To enhance readability and reproducibility we have asked and received feedback from members of the public and the scientific community. This feedback has provided us with insight into how to clarify the representation of analyses in the manuscript. Neither patients nor the public were involved in the design, conduct, reporting or dissemination plans of our research.

## 3. Results

### 3.1. Search results

**Fig 1:**
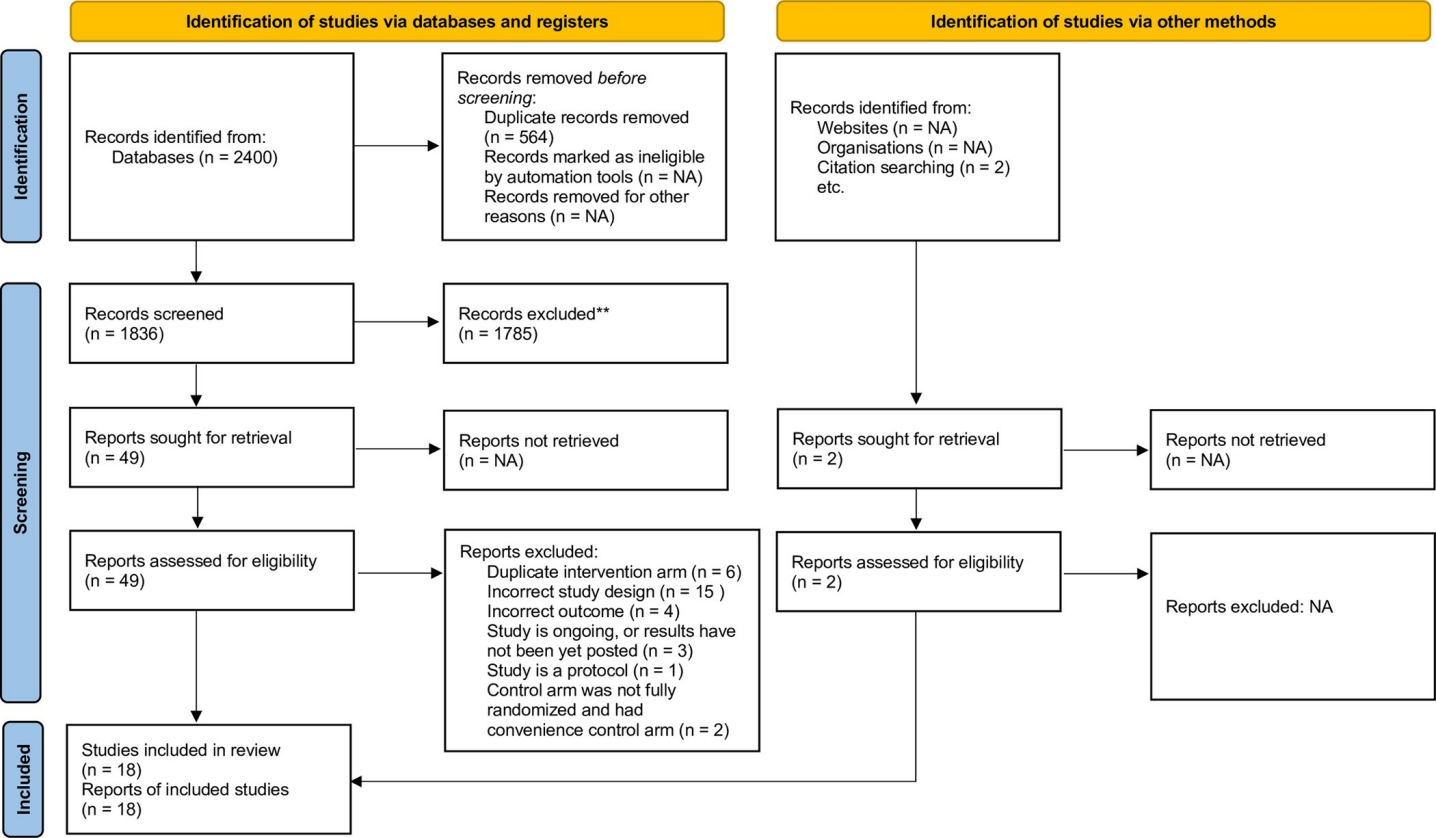
Prisma flow diagram of included articles.

Our search resulted in 2,400 unique publications. After the review, we retained 20 studies of face mask use. Two of the 20 studies could not allocate the control arm randomly because of local health regulations and recruited a separate (non-randomized) control arm and focused their analyses on differences between face masks [25,26]. Our final set of included studies were those interventions that had a randomized control arm. Thus, in total 18 studies met the full inclusion criteria for both evidence synthesis and meta-analysis (Fig 1).

The studies included a variety of settings: a pilgrimage in a community [27–30], university residence halls (2 studies) [19,20], health care (2 studies) [31,32], household (8 studies) [21,33–39], and general population in a community (2 studies) [22,40]. Eight of the trials were performed in a community setting [19,20,22,27–30,40] and ten of the trials were performed in a non-community setting that includes health care settings and households [21,31–39]. Eight studies included a mix of adults and children of different ages [21,33–39] and nine studies included only adults [19,20,22,27–29,31,32,40]. One study did not report the age of the study participants [30].

### 3.2. Characteristics of included studies

S1 Table summarizes the characteristics of each study. Altogether, these studies included 189,145 individuals in the intervention arm and 173,536 in the control arm. The duration of the follow-up varied from 4 days to 19 months. Seven of the 18 studies reported covariate adjusted odds ratio effect estimates [21,22,33–35,38,39]. The studies were carried out in various locations: Australia [36], Bangladesh [22], China [26,37], Denmark [40], France [33], Germany [39], Hong Kong [21,34], Japan [31], Saudi Arabia [27–30], Thailand [38], the United States [19,20,35], and Vietnam [32].

### 3.3. Assessment of intervention: Face mask use

In addition to the interventions being conducted in diverse settings (general population, university residents, pilgrimage, health care, household, community, and indoor) and age groups (if the intervention included only adults, if the intervention was a mix of children and adults), the interventions themselves also varied (S1 Table). Some interventions included information in the form of hygiene education, instructions on how to use face masks, and/or why face masks could be used [19,21,22,27–31,34], while in some others, the intervention included a weekly supply of face masks and a plastic bag for storage and daily disposal [28]. The type of face mask varied from cloth masks to medical masks with ear loops and to FFP2. Some studies included a separate hand hygiene regime (a hand sanitizer or instructions to wash hands), or a face mask only arm, or a hand hygiene with a face mask arm [19,20,30,34,35,38,39] (S1 Table).

Six studies [22,29,30,34,35,39] concluded that face masks decreased respiratory infections in the intention-to-treat analysis and additionally five other articles concluded that masks may be beneficial in against respiratory infections or when mitigating emerging pathogens [19,20,32,36,37]. One study had a follow-up length of five and nine weeks [22], two of the studies had a follow-up length of 6 weeks [19,20], while a third one had a follow-up of up to 19 months [35]. These four studies [19,20,22,35] also had the longest follow-up times among the 18 included studies (S1 Table).

### 3.4. Risk of bias across the studies

**Fig 2:**
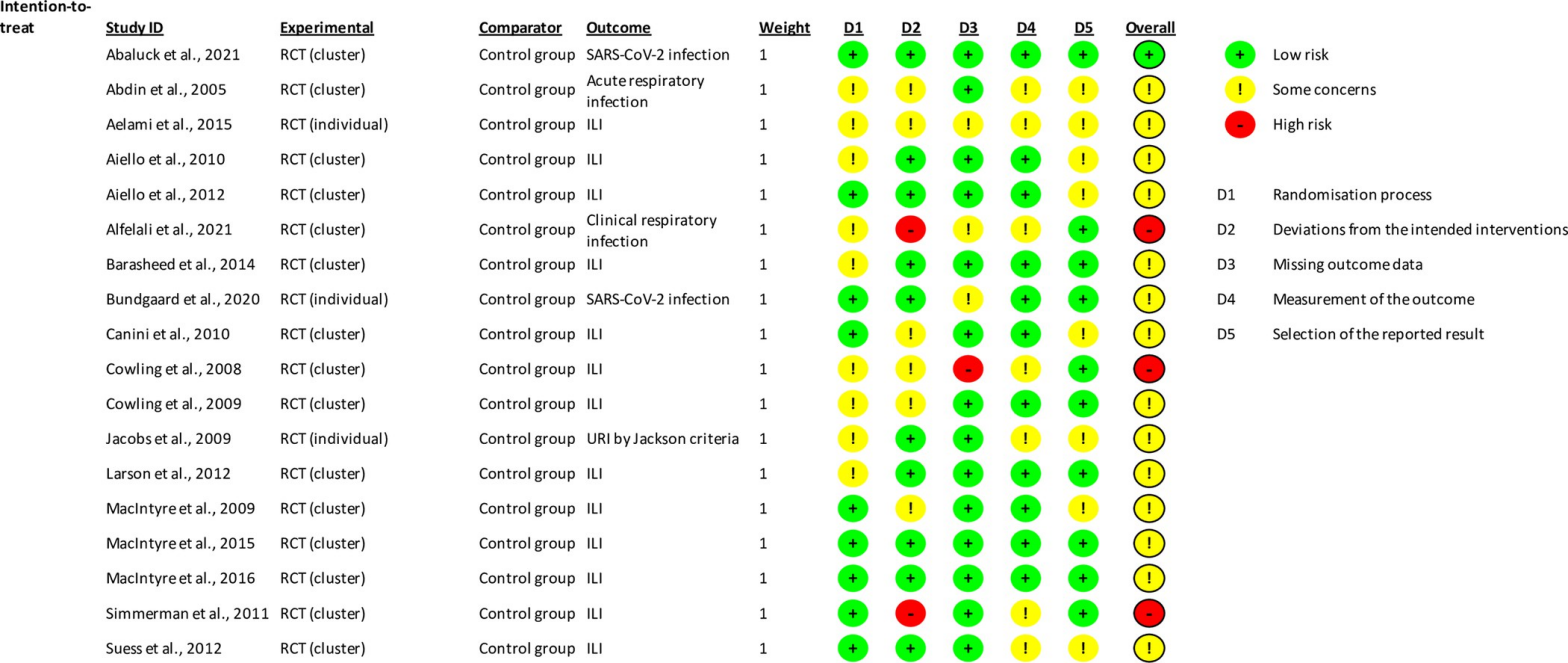
Risk of bias. Review authors’ judgement of each of the risk-of-bias item across the included studies evaluated using the Cochrane Risk of Bias 2 tool (ROB2).

Fig 2 shows a summary of the proportion of the studies that were classified at low, some concern, or high bias. The overall risk for many studies was in the middle category “some concerns”. The concerns were typically related to the randomization process [19,21,27–31,34,35], deviations from the intended interventions [21,27,28,30,33,34,36,38], and the selection of the reported results [19,20,27,30,31,33,36,39]. We did not exclude any studies based on the bias.

It was also common that that the individuals did not adhere to the assigned treatment either in the treatment or in the control arm [21,26–29,31–34,37,38] (S1 Table). In addition, one study was terminated early [33]. Almost all the trials had an increased risk of bias due to unclear or a lack of blinding. Obviously, blinding per mask use is challenging due to the visible nature of face masks.

Moreover, in some studies, the authors expressed some concerns about the lack of blinding at the stage of outcome assessment (e.g., identification of symptoms) per intervention arm (13 studies [19–21,26–29,31,32,34,35,37,40]. Similarly, it is unclear if the outcome assessment was fully blinded in any of the 18 studies (Table 1). In some studies, some of the details of randomization or random sequence generation and allocation concealment were also unclear (Table 1). Lastly, we found no evidence of a publication bias by a visual examination of the funnel plot (S1 Fig) or by an analysis based on Egger’s tests: β = 0.21 se = 0.438, p = 0.6369.

### 3.5. Face masks and respiratory infections

**Fig 3:**
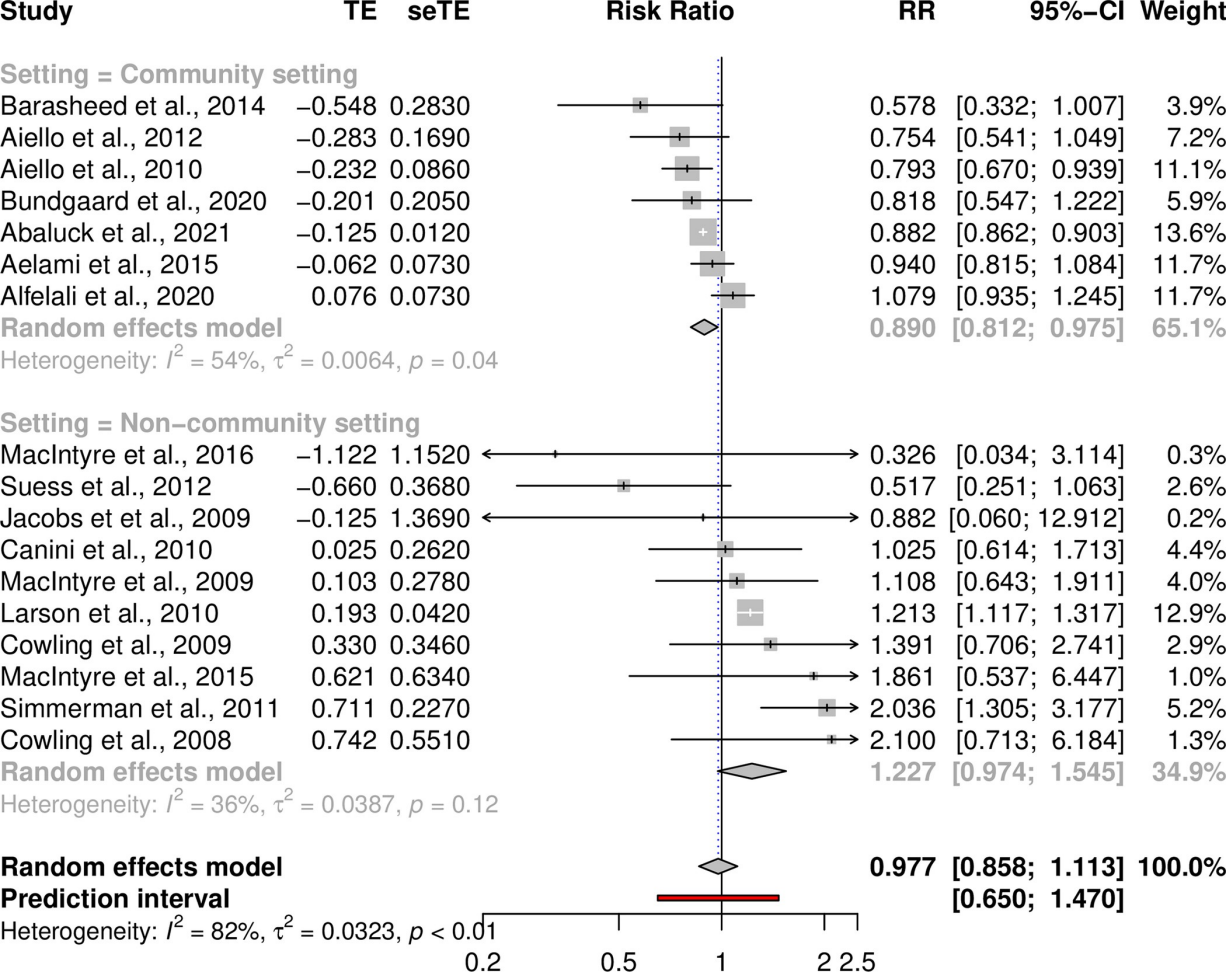
Effect of face masks across 17 studies with covariate unadjusted risk ratio estimates and subgroup analysis by community setting. Pooled effects across 17 studies of unadjusted risk ratio estimates for which the numbers of infected for each intervention arm were available using a random-effects meta-analysis model. TE = treatment effect and seTE = standard error of treatment effect, p-value = ξ^2^ is the p-value for the test of homogeneity of study-specific effect sizes.

While our meta-analysis using the covariate unadjusted risk ratio estimates found no statistically significant association between a face mask intervention and reduced respiratory infections over all studies and subgroups RR = 0.9772 [0.8582–1.1128], p = 0.728, I^2^ = 81.6%, p-heterogeneity < 0.0001, Fig 3), our subgroup analysis revealed that a face mask intervention reduced respiratory infections in a community setting (RR = 0.890 [0.812–0.975], p = 0.0125, I^2^ = 54.0%, p-heterogeneity = 0.0422, Fig 3) and when the intervention group consisted only of adults (RR = 0.8795 [0.7861–0.9839], p = 0.0249, I^2^ = 49.0%, p-heterogeneity = 0.0560, S2 Fig). The results of our other subgroup analyses (hospital, household, or source control) were not statistically significant. Moreover, our meta-regression results using the adjusted odds ratios also showed between-setting heterogeneity and the effects were affected by non-compliance when controls also used masks (S2 Table). This result is in line with the general notion of infection transmission being context, setting, and population dependent.

Our systematic review showed that the studies had baseline differences after randomization. Furthermore, the baseline differences and the between-setting heterogeneity were reflected in high I^2^ heterogeneity estimate values and low heterogeneity p-values in our analyses using covariate unadjusted relative risk effect estimates (Fig 3). Thus, to account for the baseline differences in our meta-analysis, we performed analyses using the covariate adjusted odds ratio estimates from those studies that had accounted for the baseline differences between the study arms.

**Fig 4:**
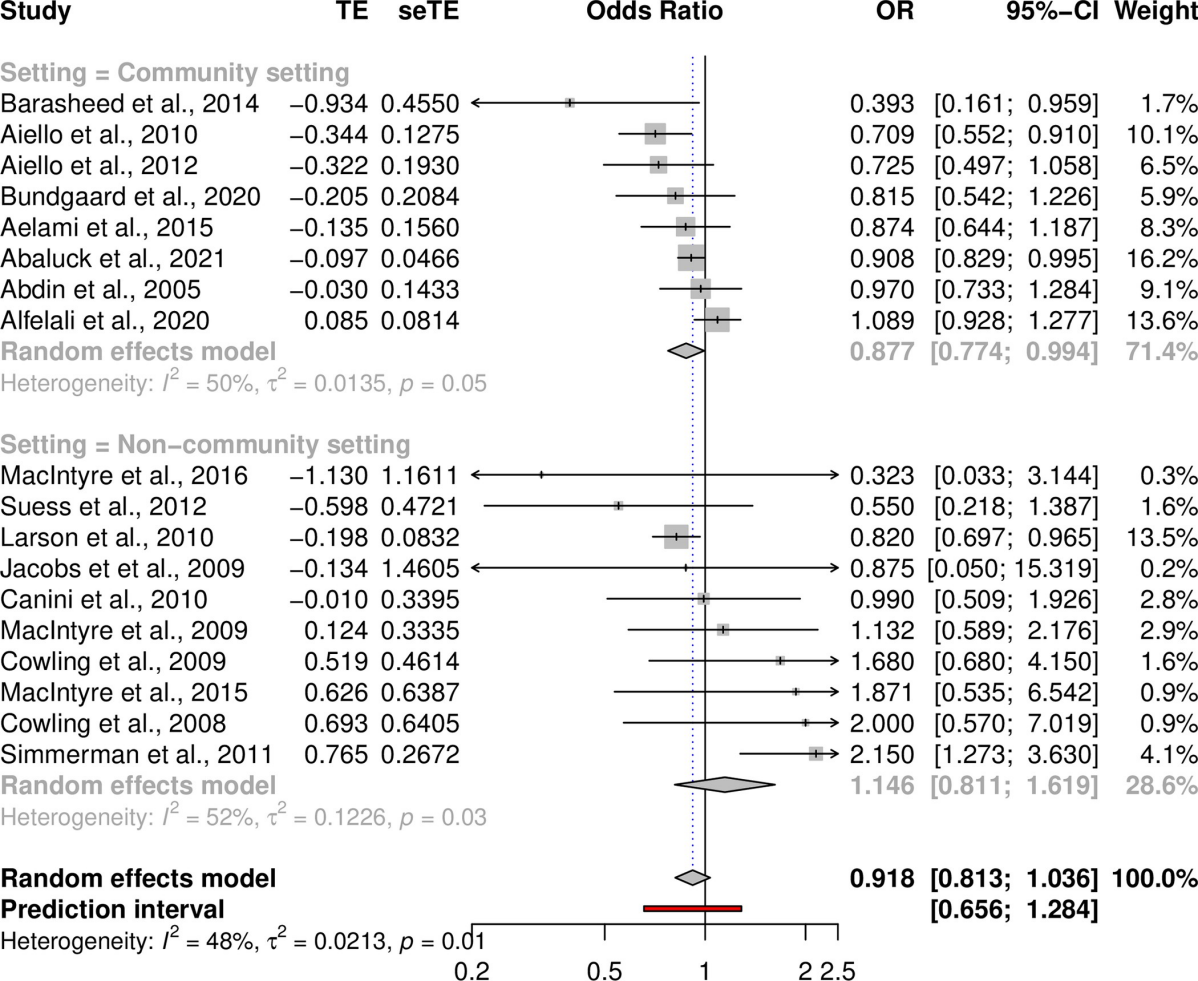
Effect of face masks across 18 studies using covariate adjusted odds ratios. Pooled effects across all 18 studies using a random-effects meta-analysis model. Seven of the 18 studies provided data for adjusted odds ratio estimates. For those studies that did not report adjusted odds ratio estimates we calculated unadjusted odds ratio estimates using the numbers of infected reported in the paper. TE = treatment effect and seTE = standard error of treatment effect, p-value = ξ^2^ is the p-value for the test of homogeneity of study-specific effect sizes.

Our analysis using the adjusted odds ratio estimates (when available) did not find a statistically significant effect of a face mask intervention (OR = 0.9177 [0.8132–1.0356], p = 0.1637, I^2^ = 48.4%, p-heterogeneity = 0.0115, Fig 4). Similarly to our results from the subgroup analysis based on the covariate unadjusted risk ratios, we found that face mask intervention reduced respiratory infections in a community setting (OR = 0.8770 [0.7736–0.9942], p = 0.0402, I^2^ = 50.1% p-heterogeneity = 0.0506) (Fig 4). However, in the setting focusing on adults only the results were not statistically significant (OR = 0.8822 [0.7692–1.0116], p = 0.0728, I^2^ = 47.5% p-heterogeneity = 0.0548).

In addition to the substantial differences resulting from unadjusted versus covariate adjusted estimates, it is also possible that individual studies may also bias the estimates of the meta-analysis. Thus, we performed a meta-analysis through a leave-one-out analysis to examine if a particular study causes a systematic association toward a given direction (S3 Fig for risk ratios). The effect sizes were all systematically at RR < 1 but not statistically significant when any single study was left out. Furthermore, when using covariate adjusted effect estimates our leave-one-out analysis found that one study, [38], biased the results towards a null effect–a face mask intervention did reduce respiratory infections (OR = 0.8892 [0.8061–0.9810], p = 0.0192 I^2^ = 28.8%, p-heterogeneity = 0.1282, S4 Fig) when study [38] was excluded from the analysis. Moreover, the trial in [38] coincided with a mask and hand washing campaign in the study location (Thailand) and there was higher level mask and handwashing in the control arm and an additional increase in reported symptoms in the study arm with combined hand washing and face mask intervention.

Furthermore, one of the largest studies, [28], had a significant level of not adhering to protocol (non-compliance) in the control arm, with 49% of the controls using face masks. In addition, the second largest study, which had the longest follow-up time, had a substantial difference in rates of infection and benefit of masks when measured as secondary attack rates by intervention group [35]. We were in contact with the author of the study but could not explore the possible causes because we did not have access to the secondary transmission values or covariates.

In 11 of the studies the intervention consisted solely of face mask use, while in seven articles the intervention also included guidance on appropriate hand hygiene together with the face mask use. This subgroup analysis for the face mask with hand hygiene guidance does not find support for a face mask intervention (OR = 0.8910 [0.6815 − 1.1649], p = 0.3987, I^2^ = 66.5%, p-heterogeneity = 0.0065). However, in this subgroup analysis we find that a face mask intervention combined with hand hygiene reduced respiratory infections (OR = 0.7968 [0.7038–0.9021], p = 0.0003, I^2^ = 0%, p-heterogeneity = 0.4366) when [38] was excluded from the analysis.

### 3.6. Number needed to treat

We approximate the effect of masks on population health by exploring the number needed to treat (NNT), that is, how many individuals need to wear a mask to prevent one person from contracting a respiratory infection. The number needed to treat depends on the number of infections at the population level. If there are few infections, a larger number of mask users will be needed to prevent one infection. Also, the effect is context-specific and depends on the baseline risk, which increases during active epidemics. Importantly, each prevented infection with an exponentially spreading disease will have a multiplicative effect, so that small changes in R0 may contribute to a large number of cases prevented. Thus, a combination of exponential growth and potential of NPIs to reduce R0 makes infectious diseases like COVID-19 different from noncommunicable diseases.

Based on the results from our meta-analysis and assuming a low baseline risk of 0.01 and OR = 0.88 in the community setting, the NNT is 841. If the baseline risk is higher, 0.1, the NNT is 92. For adults, our results indicate RR = 0.89, which corresponds to the NNT of 910 if the baseline risk is 0.01 and that of 91 for the baseline risk of 0.1.

## 4. Discussion

### 4.1. Main findings

In this systematic review and meta-analysis of 18 randomized controlled trials, we examined whether face mask intervention can prevent respiratory infections. Eleven out of 18 articles either directly supported the use of masks or concluded that masks may be beneficial against respiratory infections or in when mitigating emerging pathogens. In addition, the analysis based on covariate adjusted risk ratio estimates showed that face mask intervention at the community level and for adults could decrease respiratory infections. However, the remaining heterogeneity even in subgroup analyses indicated a large variation across the studies that needs to be taken into account both in the interpretations of the results and when designing future research on face masks; for example, based on the evidence one cannot conclude that a face mask intervention is not effective for younger individuals too.

We find that face mask intervention reduces respiratory infections in particular when intervention is conducted in a community setting and face masks are combined with appropriate hand hygiene. Our results are in line with the current recommendations by the public health experts that face mask recommendations are most efficient when used together with appropriate hand hygiene and other NPIs. It is worth noting that, despite the relatively large between-study heterogeneity and small effect sizes in the individual articles, the findings did support use of face masks. Therefore, these findings, together with the mounting other evidence, suggest that face masks may be considered as a useful NPI for respiratory infections, including COVID-19.

### 4.2. Quality of evidence

We found 18 randomized controlled trials that had assessed whether a face mask intervention can mitigate the risk of respiratory infections. Other earlier studies have been conducted using case-control settings or with masks with a strong filtering capacity [8]. An earlier systematic review and meta-analysis has investigated the effect of face masks by including randomized controlled trials, case-control studies, and cohort studies in health care and non-health care settings [16] and another study has focused on the efficacy of cloth masks [15]. The findings from our systematic review and meta-analysis are in line with the conclusions of those earlier meta-analyses conducted in different settings.

While the intention-to-treat analysis yields an unbiased estimate of the efficacy of the face mask intervention, its magnitude is biased downwards relative to the actual treatment effect of face masks. While the overall quality of the earlier trials is solid, we find that there were biasing factors across the studies, including a compliance bias either because of low compliance in the face mask intervention arm or the use of face masks in the control arm, which may bias the estimates towards the null hypothesis. This is also confirmed by our sensitivity analysis.

In addition, because the effect with hand hygiene seems to be stronger than with mask use alone, one might conclude that hand hygiene is driving the association while mere face masks do not protect from respiratory infections. First, in the context of SARS-CoV-2, the main mode of its spread is through airborne transmission [3–7]. Second, while masks have been shown to be effective in themselves, their impact and, therefore, efficacy is greatest when combined with other protective measures [8]. Our study indeed found that the effect of masks was further enforced when combined with complementary measures, such as improved hand hygiene.

Furthermore, other complementary measures for disease control of airborne infectious disease, such as physical distancing measures, can have an impact on the spread of diseases and the number of particles in the air and, hence, could also be combined as a prevention tool together with face masks. Indeed, in one review [13], the estimated number needed to use masks to prevent one infection ranged from three (N95 masks) to six (face masks), and the number is higher still when the infection risk is low to start with. Clearly, these NNTs are only approximations since the reproduction number R differs between pathogens. Similarly, if there are no active infections, the NNT will be infinite: no infections can be prevented as none are present in the population.

With these limitations in mind and assuming a low baseline risk of 0.01, we calculated that, for respiratory infections, the NNT might range from 92 to 910. To put this into context, let us presume that, in a metropolitan area with a population of 1 million, 30% of the residents use face masks. With NNT = 455, this might prevent 600 respiratory infections. This effect size is comparable to the NNT of pharmaceuticals. For example, the NNT for selective serotonin reuptake inhibitors (commonly known as SSRI), tricyclic depression medications or therapy have a relatively low NNT ranging from 4 to 11 [41,42]. In comparison, condoms, a non-pharmacologic intervention to prevent adolescent pregnancies, have a NNT of 21 [43]. Finally, the NNT for statins, some of the most widely prescribed drugs in primary prevention of major vascular events (CVD risk 5–10% within 5 years), ranges from 67 to 170 and is of a similar scale to that of face masks [44]. Furthermore, each prevented infection with an exponentially spreading disease will have a multiplicative effect; small changes in R0 may contribute to a large number of cases prevented. Exponential growth, and the possibility to reduce R0 makes infectious diseases like COVID-19 different from diseases that are not transmitted.

In sum, our analysis shows that studies focusing on a community setting and in the studies in which hand hygiene was assessed together with mask use, reductions in infections with multiplicative protective measures were seen. These results support the use of face masks in preventing respiratory infections and, hence, the WHO guidelines that recommend the use of face masks together with physical distancing and hand hygiene to control the spread of COVID-19.

### 4.3. Limitations

Our analysis has the following three limitations. The first is that the populations studied here had residual heterogeneity: because respiratory infections are time- population and exposure-dependent, it is possible that differences in follow-up times and in symptom assessments (influenza-like illness, respiratory illness, or COVID-19) have affected the power to detect associations. Second, while all the articles reported the numbers in the intervention and control arms, we did not have access to the original data with covariates and thus could not perform a full analysis with all the covariate adjusted odds ratio estimates or by adjusting within-study variables. As discussed, we also did not have access to the covariate unadjusted values for the secondary transmission (source control) in one of the articles [35]. Our study may also suffer from the potential bias caused by intracluster correlation as the clustered nature of original studies had not been addressed in the unadjusted effect estimates that were provided by the studies. As a work-around, we performed a meta-analysis including the within-study covariate adjusted odds ratios. However, this method comes with limitations of its own as, in practice, no studies had exactly the same covariate definitions, which biases the estimates.

Third, the mask types and instructions for mask use together with follow-up times and type of study population varied by study, which likely increases between-study heterogeneity. We accounted for some of the biases through a subgroup analysis by age, setting, and non-compliance.

### 4.4. Conclusions and future implications

This systematic review and meta-analysis of 18 randomized controlled trials across different exposure settings and age groups provides support for the public health policy of face mask use to limit the spread of respiratory infections, such as COVID-19. Our analysis suggests that face masks can decrease the probability of spreading and contracting a respiratory infection and can be particularly useful when combined with other personal protection methods, especially at the community level. However, compliance has a large impact on the effectiveness of face masks: many studies were subject to contamination bias thus affecting the efficacy of the intervention, that is when also the controls used masks or when the intervention group did not comply with mask use leading to a downward biased effect of treatment receipt and efficacy. Clearly, an unworn mask cannot protect people from respiratory infections.

Recommendations and clear communication about the benefits of face masks and how to use them should be given by policymakers to provide individuals with the tools to protect themselves and others from respiratory infections. Mask wearing can ultimately reduce the negative consequences of respiratory disease pandemics.

## Supporting information

S1 Checklist PRISMA 2020 abstract checklist. (DOCX)

S2 Checklist PRISMA 2020 checklist. (DOCX)

S1 Fig Funnel plot odds ratio estimates. (PDF)

S2 Fig Risk ratio estimates in adults. (PDF)

S3 Fig Leave-one-out analysis using risk ratio estimates. (PDF)

S4 Fig Leave-one-out analysis using odds ratio estimates. (PDF)

S1 Table Description of individual studies that are part of the manuscript. (ZIP)

S2 Table Meta-regression analysis. (DOCX)

S1 File R code for face mask paper. (ZIP)
