# Correction: Face masks to prevent transmission of respiratory infections: Systematic review and meta-analysis of randomized controlled trials on face mask use

**DOI:** 10.1371/journal.pone.0320226

**Published:** 2025-03-04

**Authors:** Hanna M. Ollila, Markku Partinen, Jukka Koskela, John Borghi, Riikka Savolainen, Anna Rotkirch, Liisa T. Laine

After this article [1] was published, concerns were raised about the study methodology and reporting. The *PLOS One* Editors followed up on the issues raised and consulted with a member of the *PLOS One* Editorial Board, an external reviewer, and a statistical reviewer. The *PLOS One* Editors consider that the issues raised do not affect the results and conclusions of the article and are addressed with the publication of this Correction to inform readers about the incorrect inclusion of one study in the odds ratio meta-analysis, and provide additional information and clarifications. The conclusions of [1] remain unchanged.

## Study inclusion error

A concern was raised about the inclusion of Abdin et al. [2] in the meta-analysis for those analyses that used odds ratio, since [2] reports the odds ratio for infection of compliance vs. non-compliance of mask wearing, and it is not possible to calculate the odds ratio for face mask intervention vs. control from the data reported. This error does not affect the primary analyses of [1] that were performed with risk ratio. Moreover, the results of odds ratios without [2] was already reported in the leave-one-out analysis as part of the original article. Here, the authors provide results of the analyses that used odds ratio with [2] excluded (provided with this notice as S1–S4 Corrections). A statistical reviewer assessed these data and advised that [2] should not have been included in the analyses that used odds ratio, but this did not affect either the results of the analyses, or any of the conclusions of [1].

## Amendments to PROSPERO

Concerns were raised about changes in PROSPERO (CRD42020205523) on 23 September 2022–after the manuscript was accepted–that were not discussed in the manuscript. Here, the authors highlight and justify changes made to the registry entry. An external reviewer concluded that the amendments were justified, but should have been acknowledged in the published manuscript, as recommended by PRISMA guidelines.

A complete list of changes is provided below:

- Post-acceptance edit included adding citation to the accepted manuscript.
- The review title was changed to match the changes suggested by the reviewers.
- The anticipated completion date was changed to 30/09/2022.
- Review team members and their organizational affiliations were changed to match their new affiliations. Also, because reviewers asked authors to conduct additional analyses, including doing risk of bias using the new Cochrane Risk of Bias 2 (RoB2) tool, the roles of authors were updated to meet these new tasks.
- Grant number(s) were changed: Added information of a new grant that was awarded during the reviewer process and removed a grant number that did not provide funding for the study.
- Updated the list of collaborators as some of them moved to work in a different institute.
- Review question: Reviewers suggested to update the review question, and we updated the question and harmonized across the abstract, methods and PROSPERO.
- Searches: By reviewer request, the search strategy was changed to include also the most recent publications.
- Search strategy: This was updated to be an exact match with the updated search strategy (due to reviewer request).
- Domain being studied: Was slightly edited to use consistent terminology in the field.
- Interventions, exposures: Was edited to use consistent terminology in the field.
- Main outcomes: Adjusted odds ratio was added due to reviewer request.
- Measures of effect: Was updated to match with the reviewer request to add adjusted odds ratio measure.
- Data extraction: Was updated to match the description provided in the revised manuscript after incorporating reviewer requests.
- Risk of bias assessment: Was updated to match with RoB2 due to reviewer request.
- Strategy for data synthesis: Was updated to match the description provided in the revised manuscript after incorporating reviewer requests.
- Analyses of subgroups or subsets: Health care, community, and household settings were added due to reviewer comments.
- Country: Added country USA.

## Additional information

The authors provide the following additional information and clarifications:

- The authors here provide a detailed breakdown of the RoB2 assessment in S5 Correction. The authors acknowledge Dr. Tuuli Hietamies, Stanford University, for critical discussions on the use of RoB2. No specific errors were observed in the RoB2 analysis during the follow-up of this case.
- The meta-analysis in [1] included only clinical definition results, and not laboratory-confirmed results from included studies. This may miss asymptomatic cases and asymptomatic transmission and does not distinguish between specific pathogens. However, this approach has the advantage of catching symptomatic cases across pathogens, not just for one specifically tested pathogen.
- MacIntyre et al. 2009 [3] and MacIntyre et al. 2016 [4] were excluded from the adult subgroup analysis because this subgroup analysis excluded studies that also included children in the household, and the effect of the intervention on outcomes/infections in [3] and [4] were reported in children and adults.
- A list of reports excluded in the full text review is not provided in [1]; this is now provided here (S6 Correction). The authors clarify that to be included in [1], studies must meet the inclusion criteria reported in Table 2, which stipulate that the study design must be a randomized control trial for which both face mask intervention and no-face mask control groups are allocated at random, and the study must measure respiratory infection. Linked reports were handled as duplicates.
- The code used for the meta-regression analysis is not included in [1], as it was part of the submission files but it was not included in the published files; the Stata program code underlying this analysis is provided here (S7 Correction).

## Supporting Information

S1 Correction Forest plots for aOR community subgroup. Re-analysis of the community setting subgroup of Fig 4 without Abdin et al.
(PDF)

S2 Correction Forest plots for aOR adults subgroup.
Re-analysis without Abdin et al. of the results reported in the third paragraph of section 3.5. Face masks and respiratory infections.
(PDF)

S3 Correction Funnel plot.
Re-analysis of S1 Fig without Abdin et al. Egger’s test without Abdin et al: beta = 0.21, se = 0.445, p =  0.6320.
(PDF)

S4 Correction Leave-one-out analysis odds ratios.
Re-analysis of S4 Fig without Abdin et al.
(PDF)

S5 Correction Detailed risk of bias assessment.
(XLSX)

S6 Correction List of excluded full-text reports. (XLSX)

S7 Correction Meta-regression code. Stata program code underlying the meta-regression in S2 Table.
(ZIP)

## References

[1] Ollila HM, Partinen M, Koskela J, Borghi J, Savolainen R, Rotkirch A, et al. Face masks to prevent transmission of respiratory infections: Systematic review and meta-analysis of randomized controlled trials on face mask use. PLoS One. 2022;17(12):e0271517. doi: 10.1371/journal.pone.0271517 36454947 PMC9714953

[2] Abdin EZ, Choudhry A, Al-Naji D. Effect of use of Face mask on Hajj related Acute Respiratory Infection among Hajjis from Riyadh—A Health Promotion Intervention study. Saudi Epidemiol Bul. 2005;12(4):27–28.

[3] MacIntyre CR, Cauchemez S, Dwyer DE, Seale H, Cheung P, Browne G, et al. Face mask use and control of respiratory virus transmission in households. Emerg Infect Dis. 2009;15(2):233–41. doi: 10.3201/eid1502.081167 19193267 PMC2662657

[4] MacIntyre CR, Zhang Y, Chughtai AA, Seale H, Zhang D, Chu Y, et al. Cluster randomised controlled trial to examine medical mask use as source control for people with respiratory illness. BMJ Open. 2016;6(12):e012330. doi: 10.1136/bmjopen-2016-012330 28039289 PMC5223715

