# Correction: Face masks to prevent transmission of respiratory infections: Systematic review and meta-analysis of randomized controlled trials on face mask use

**DOI:** 10.1371/journal.pone.0325466

**Published:** 2025-05-30

**Authors:** The PLOS One Editors

This article [1] was previously corrected in [2] to address concerns including incorrect inclusion of one study [3] in the odds ratio meta-analysis; however, the Correction [2] did not include a reanalysis of the meta-regression excluding Abdin et al. [3]. Upon follow-up, the first and last authors provided a revised meta-regression (S8 Correction). Based on the recommendation of a statistical reviewer, who reviewed the reanalysis, the authors note that the meta-regression was originally run as an additional sensitivity analysis and can be interpreted as exploratory on its own, given the limited number of observations. They further stated that the conclusions of [1] remain unchanged.

## Supporting information

S8 Correction Meta-regression analysis. Re-analysis of the meta-regression without Abdin et al.
(PDF)
