# Supplementary figures and images for: Face masks to prevent transmission of respiratory infections: Systematic review and meta-analysis of randomized controlled trials on face mask use
Source: PLoS One. 2022 Dec 1;17(12):e0271517. doi: 10.1371/journal.pone.0271517 (PMC9714953; doi:10.1371/journal.pone.0271517)

# Contour-enhanced funnel plot

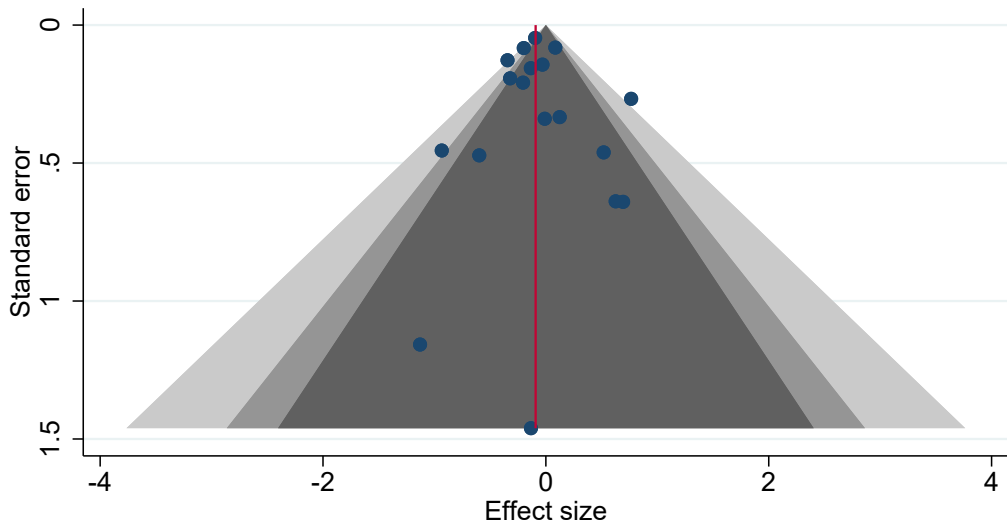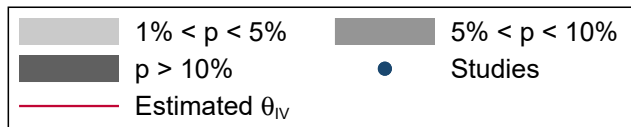

Supplement: S1 Fig — (PDF) [file pone.0271517.s003.pdf]

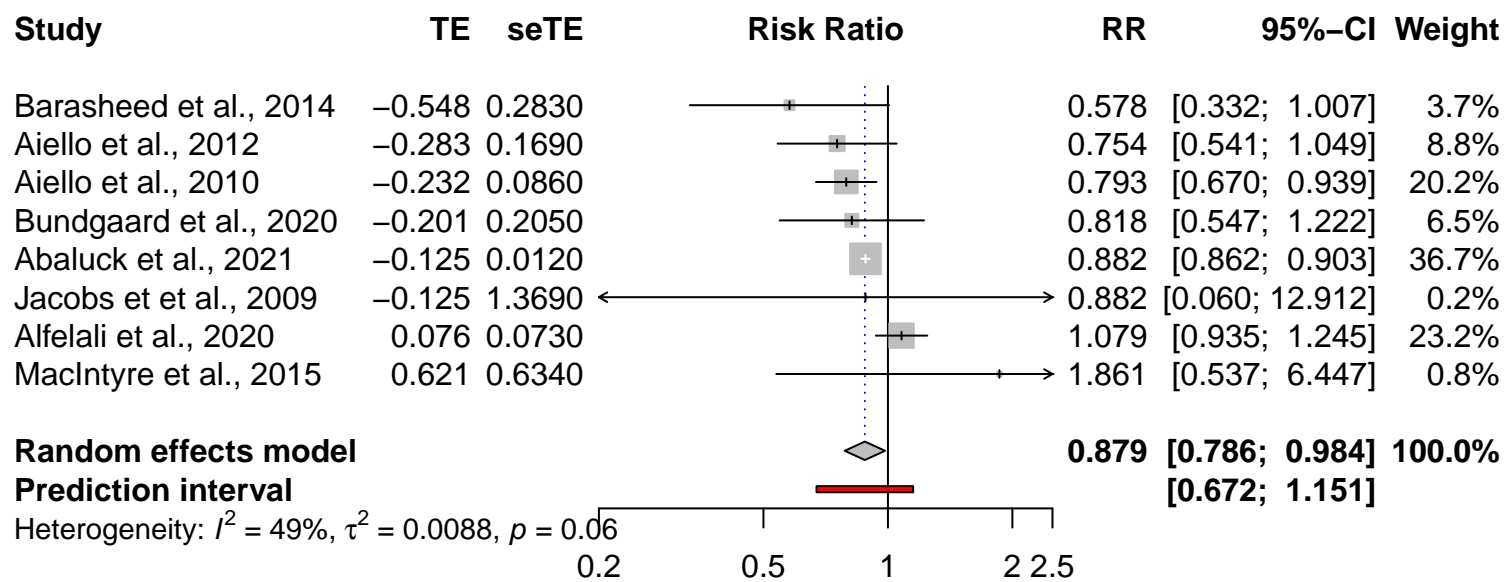

Supplement: S2 Fig — (PDF) [file pone.0271517.s004.pdf]

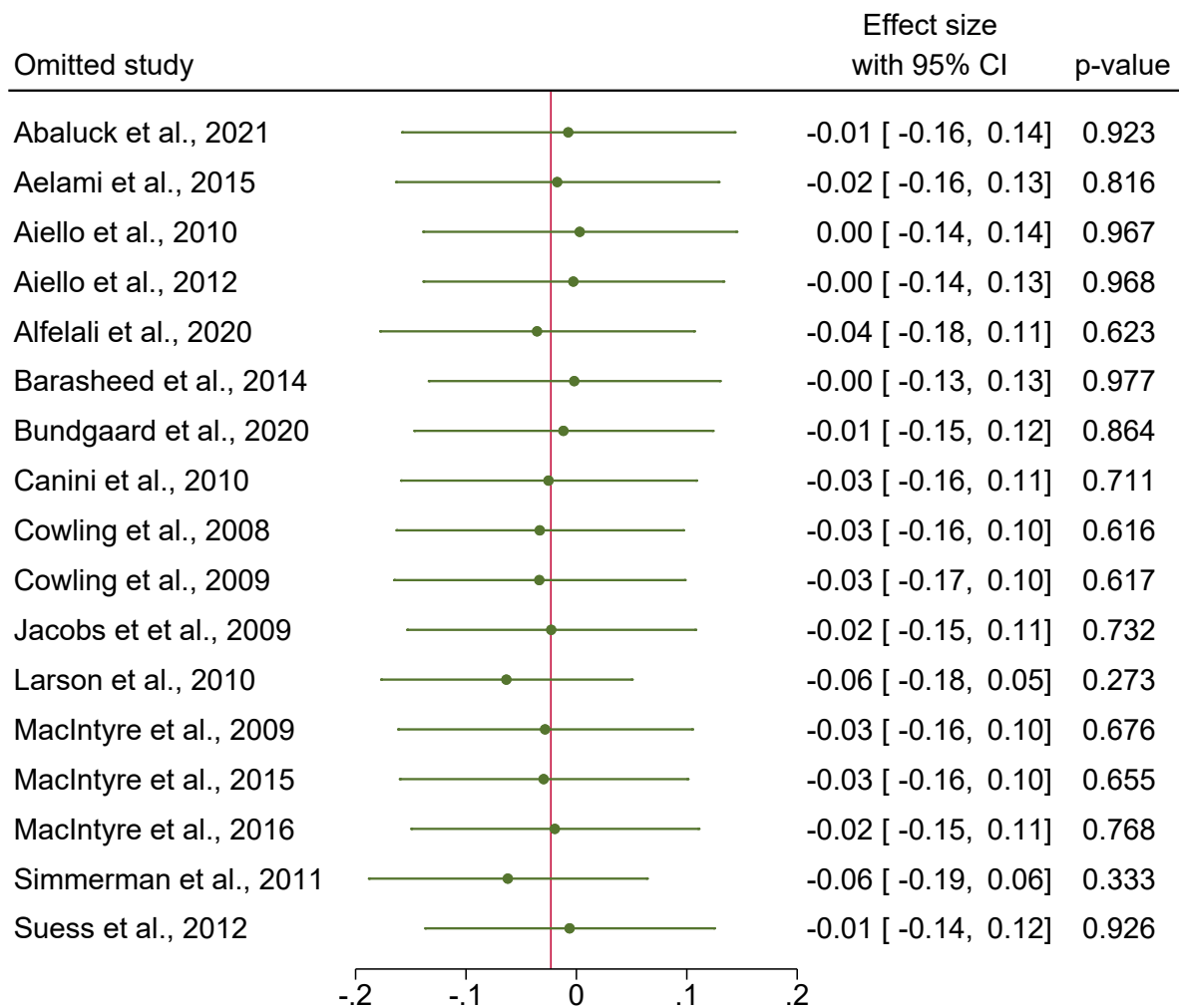

Random-effects DerSimonian–Laird model

Supplement: S3 Fig — (PDF) [file pone.0271517.s005.pdf]

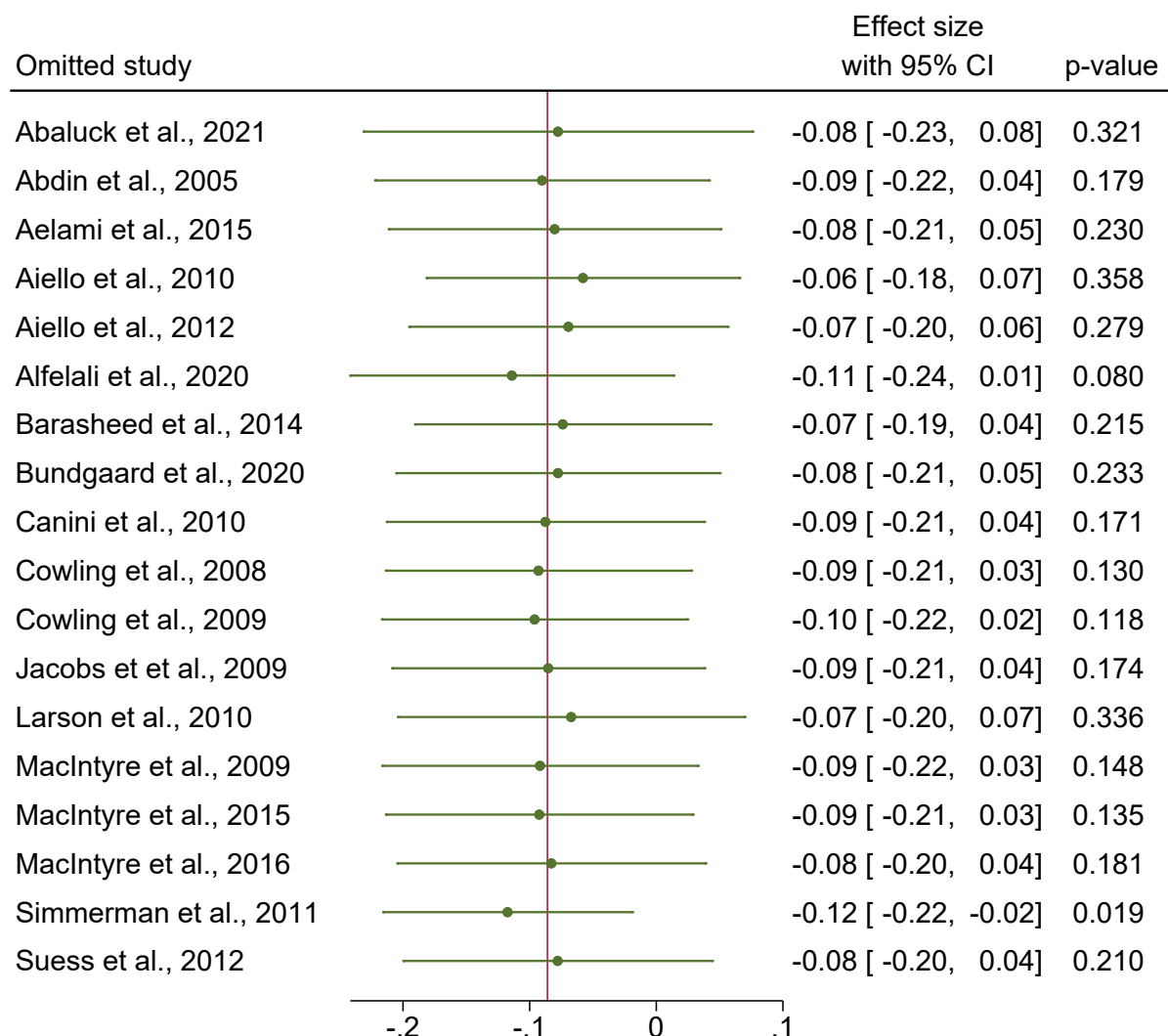

Random-effects DerSimonian–Laird model

Supplement: S4 Fig — (PDF) [file pone.0271517.s006.pdf]

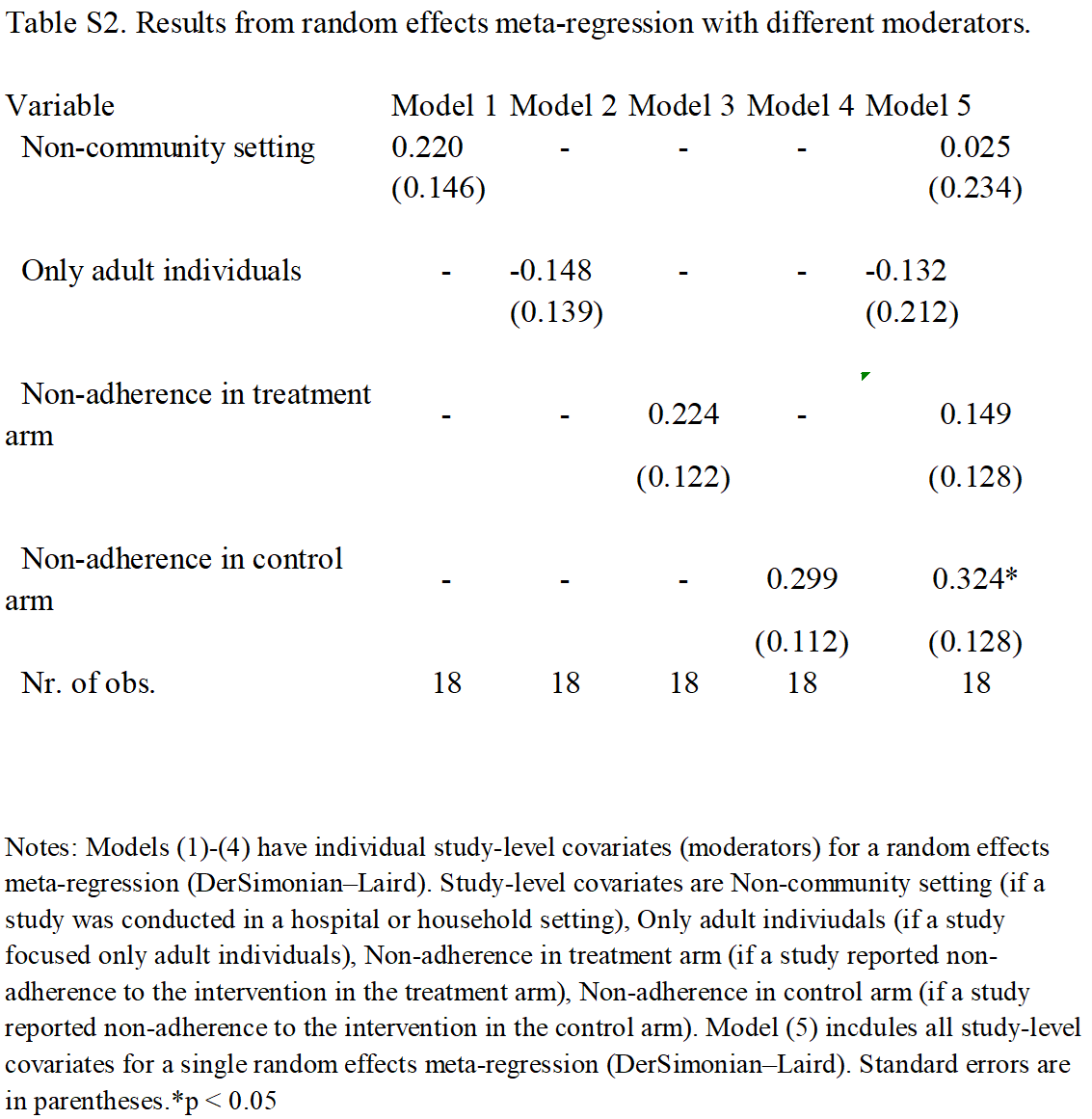

Supplement: S2 Table — (DOCX) [file pone.0271517.s008.docx]
