# Supplementary figures and images for: Correction: Face masks to prevent transmission of respiratory infections: Systematic review and meta-analysis of randomized controlled trials on face mask use
Source: PLoS One. 2025 Mar 4;20(3):e0320226. doi: 10.1371/journal.pone.0320226 (PMC11878934; doi:10.1371/journal.pone.0320226)

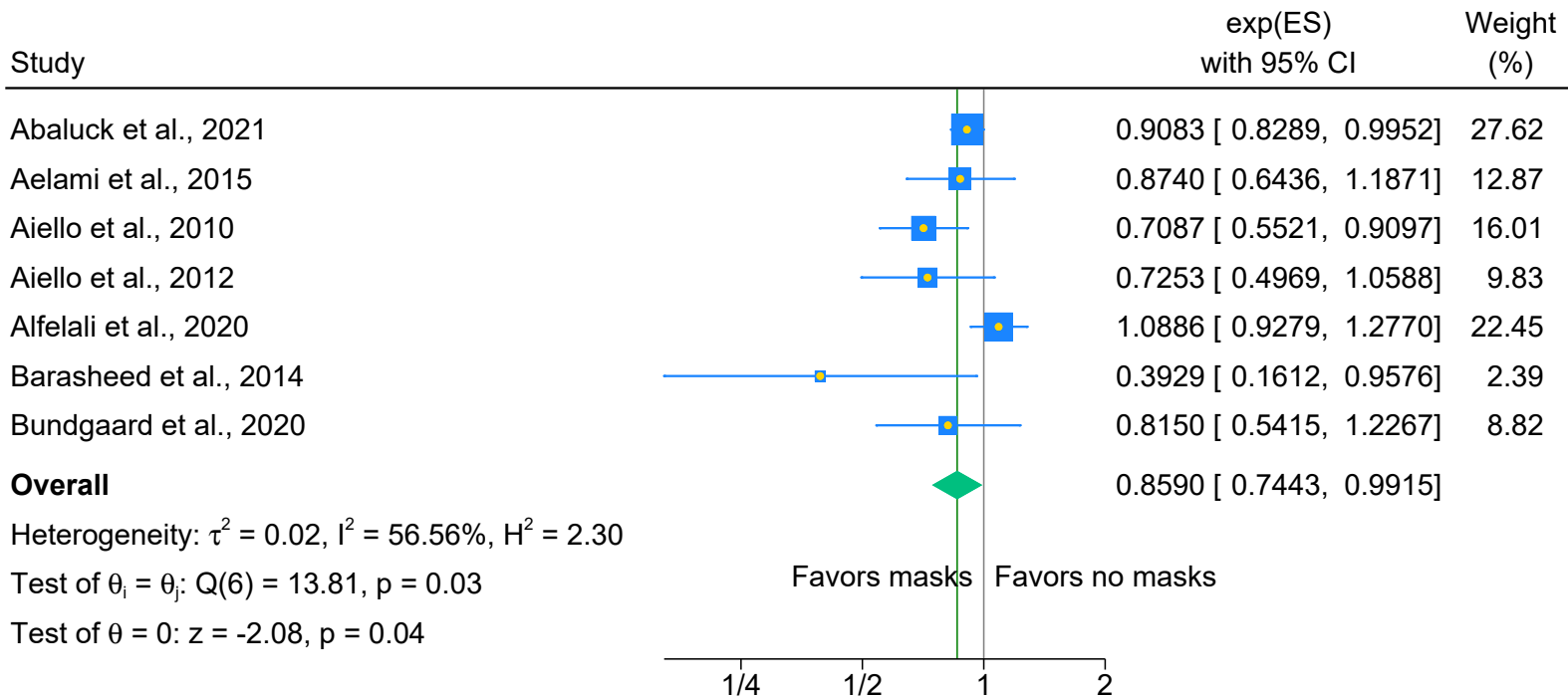

Random-effects DerSimonian–Laird model

Supplement: S1 Correction — Re-analysis of the community setting subgroup of Fig 4 without Abdin et al. (PDF) [file pone.0320226.s001.pdf]

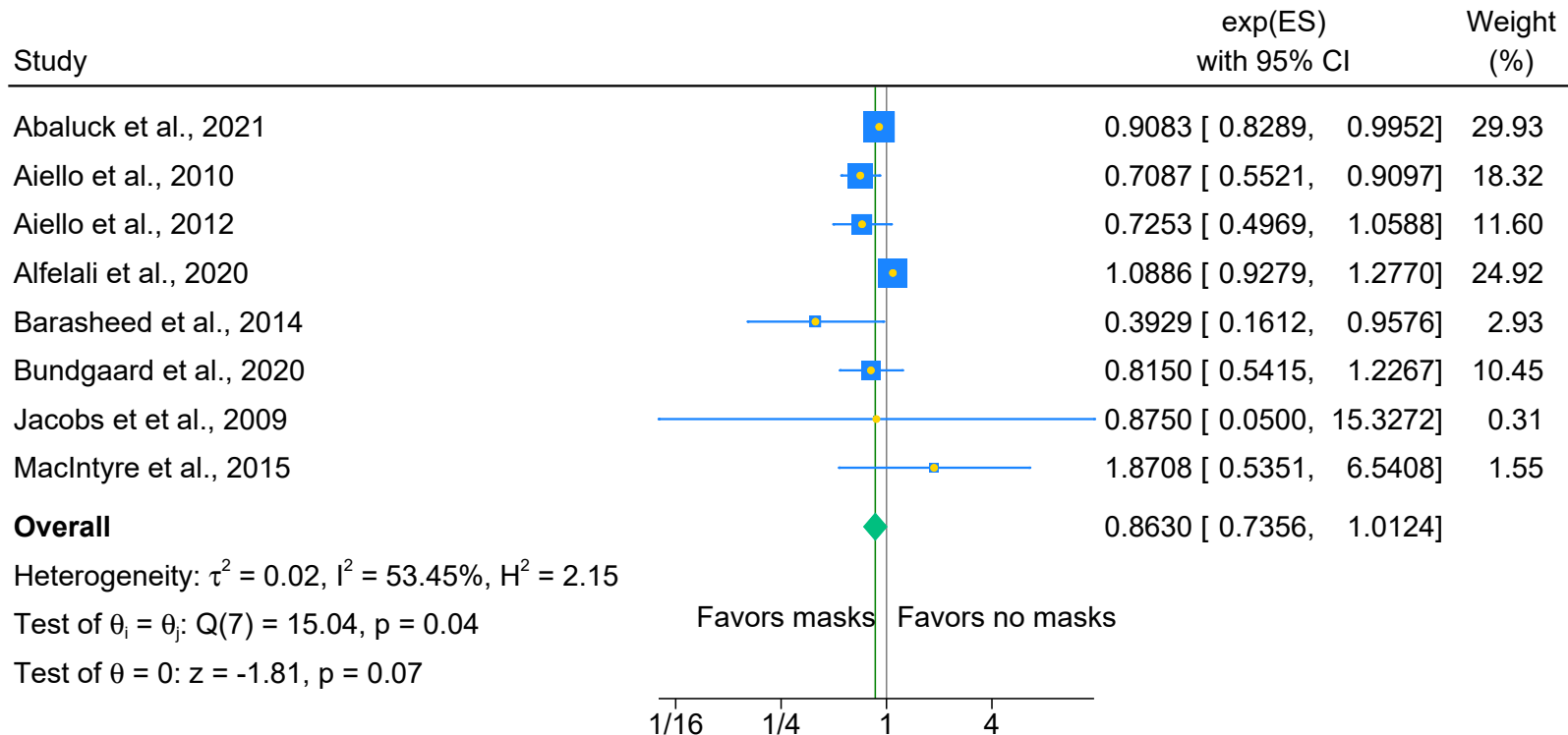

Random-effects DerSimonian–Laird model

Supplement: S2 Correction — Re-analysis without Abdin et al. of the results reported in the third paragraph of section 3.5. Face masks and respiratory infections. (PDF) [file pone.0320226.s002.pdf]

# Contour-enhanced funnel plot

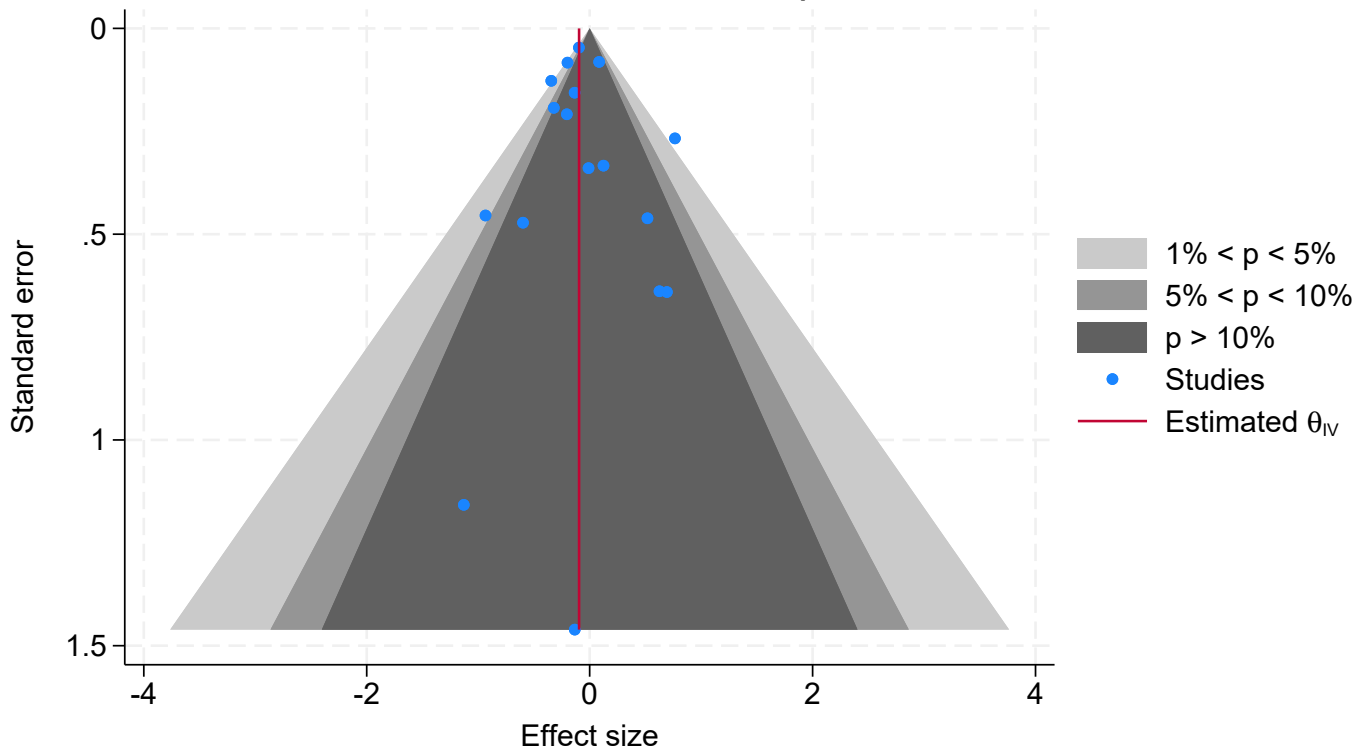

Supplement: S3 Correction — Re-analysis of S1 Fig without Abdin et al. Egger’s test without Abdin et al: beta = 0.21, se = 0.445, p = 0.6320. (PDF) [file pone.0320226.s003.pdf]

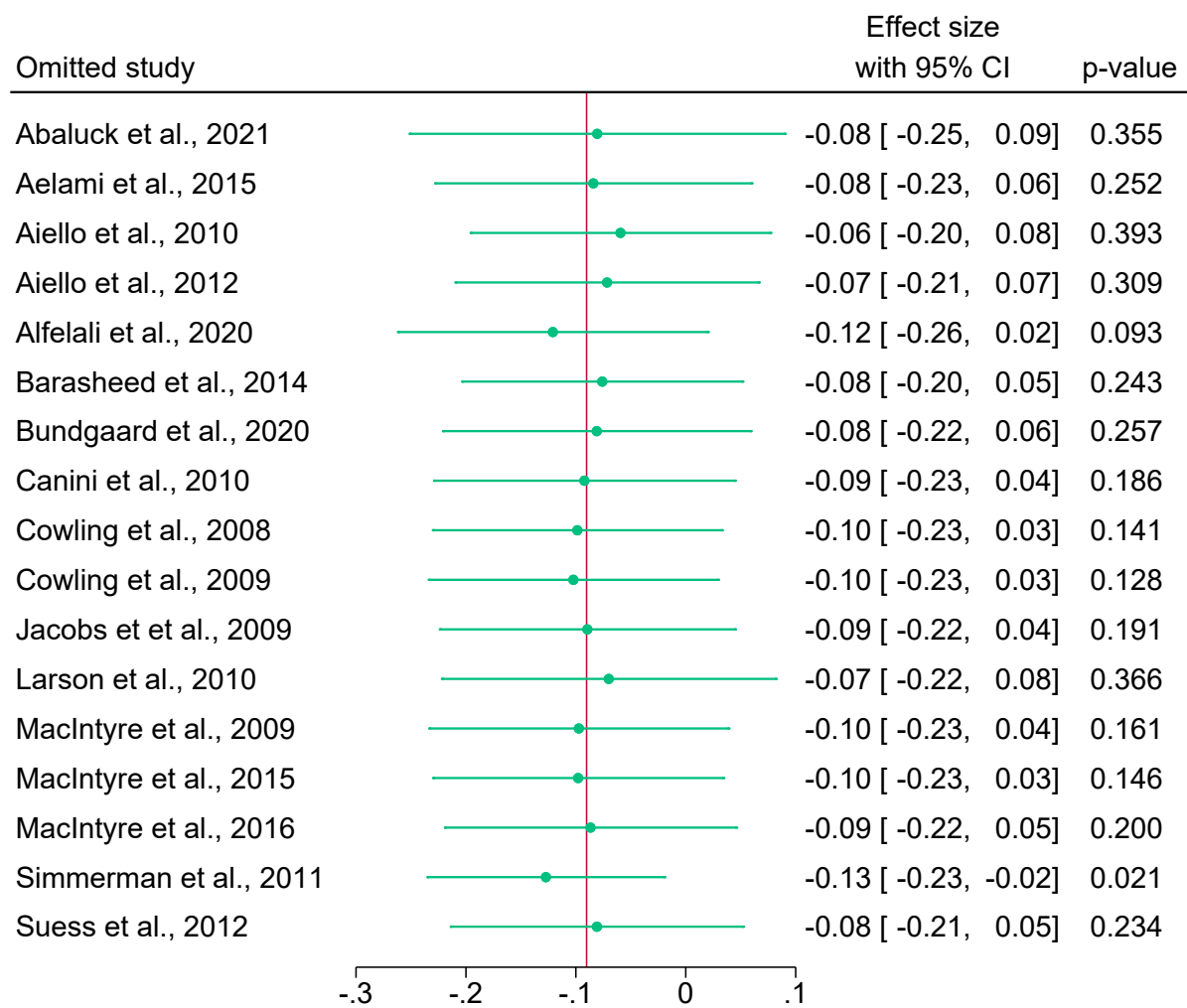

Random-effects DerSimonian–Laird model

Supplement: S4 Correction — Re-analysis of S4 Fig without Abdin et al. (PDF) [file pone.0320226.s004.pdf]
