# Supplementary material for: Correction: Face masks to prevent transmission of respiratory infections: Systematic review and meta-analysis of randomized controlled trials on face mask use
Source: PLoS One. 2025 May 30;20(5):e0325466. doi: 10.1371/journal.pone.0325466 (PMC12124534; doi:10.1371/journal.pone.0325466)
Supplement: S8 Correction — Re-analysis of the meta-regression without Abdin et al. (PDF) [file pone.0325466.s001.pdf]

Table S2. Results from random effects meta-regression with different moderators without Abdin et al. study.

Variable	Model 1	Model 2	Model 3	Model 4	Model 5
Non-community setting	0.253	-	-	-	0.060
	(0.156)				(0.259)
Only adult individuals	-	-0.179	-	-	-0.126
		(0.150)			(0.235)
Non-adherence in treatment arm	-	-	0.269*	-	0.135
			(0.130)		(0.162)
Non-adherence in control arm	-	-	-	0.359*	0.347*
				(0.129)	(0.170)
Nr. of obs.	17	17	17	17	17

Notes: Models (1)-(4) have individual study-level covariates (moderators) for a random effects metaregression (DerSimonian–Laird). Study-level covariates are: Non-community setting (if a study was conducted in a hospital or household setting), Only adult individuals (if a study focused only on adult individuals), Non-adherence in treatment arm (if a study reported non-adherence to the intervention in the treatment arm), Non-adherence in control arm (if a study reported non-adherence to the intervention in the control arm). Model (5) includes all study-level covariates for a single random effects meta-regression (DerSimonian–Laird). Standard errors are in parentheses. *$p < 0.05$
